# Longitudinal assessment of B-RAF V595E levels in the peripheral cell-free tumor DNA of a 10-year-old spayed female Korean Jindo dog with unresectable metastatic urethral transitional cell carcinoma for monitoring the treatment response to a RAF inhibitor (sorafenib)

**DOI:** 10.1080/01652176.2021.1905194

**Published:** 2021-05-05

**Authors:** Jung-Hyun Kim, Dana Hyunjung Ahn, Je-Sung Moon, Hyun-Jung Han, Kieun Bae, Kyong-Ah Yoon

**Affiliations:** a Department of Veterinary Internal Medicine, College of Veterinary Medicine, Konkuk University, Seoul, South Korea; b Department of Veterinary Internal Medicine, Konkuk University Veterinary Medical Teaching Hospital, Seoul, South Korea; c Veterinary Emergency Medicine and Critical Care, Konkuk University Veterinary Medical Teaching Hospital, Seoul, South Korea; d Department of Veterinary Biochemistry, College of Veterinary Medicine, Konkuk University, Seoul, South Korea

**Keywords:** Dog, transitional cell carcinoma, circulating cell-free tumor DNA, B-RAF V595E, vascular endothelial growth factor, sorafenib

## Abstract

Transitional cell carcinoma (TCC) is the most common malignant tumor of the canine urinary tract. In this case study, a dog with metastatic urethral TCC was treated with sorafenib. The tumor expression levels of receptor tyrosine kinase genes, including *VEGFR-1, VEGFR-2, PDGFR-α, PDGFR-β, ALK, EGFR, ErbB2*, and *B-RAF*, were analyzed. VEGFR was overexpressed in tumor tissues compared to the normal tissues. Considering the high frequency of B-RAF mutation in canine urological tumors, the *B-RAF* gene was examined, and the B-RAF V595E mutation was detected in the tumor tissue. Therefore, the antitumor effect of sorafenib, a multi-tyrosine kinase inhibitor, on unresectable metastatic urethral TCC characterized by B-RAF V595E was evaluated and circulating cell-free tumor DNA (ctDNA) was assessed for monitoring the treatment response. After the initiation of oral sorafenib therapy (4 mg/kg/day escalated to 10 mg/kg/day), the dysuria was alleviated gradually, and the patient remained stable for 3 months. During that treatment period, the patient showed various levels of changes associated with B-RAF V595E mutation in ctDNA as evident from longitudinal plasma samples after initiation of sorafenib therapy. The findings of this study suggest that ctDNA may serve as a useful non-invasive tool for monitoring the treatment response to anticancer drugs.

A 10-year-old spayed female Korean Jindo Dog weighing 24.7 kg was referred to the Veterinary Teaching Hospital at Konkuk University (Seoul, South Korea) for evaluation of a 6-month history of intermittent severe dysuria and hematuria, and unresponsiveness to oral antibiotic therapy. Physical evaluation revealed panting and normal body temperature (38.9 °C), as well as tachycardia (180 beats per minute). Complete blood count and serum chemistry tests revealed all analytes to be within the reference intervals. The thoracic radiographs showed no abnormalities. On the abdominal radiograph, a superimposed soft opacity, ventral to the sacrum and dorsal to the descending colon was observed. Transabdominal ultrasound revealed an irregular and hyperechoic change (urethral wall thickness: 10.3 mm) along the proximal part of the urethra. Further, enlarged, irregularly shaped, hyperechoic sublumbar lymph nodes were detected. Urinalysis revealed microscopic hematuria. Cytological examination of urine revealed the presence of numerous binucleated transitional cells, neutrophils, and red blood cells. The result of a urine culture test was negative. The owner of the dog elected to pursue computed tomography (CT) for further analysis of the urethral mass. An abdominal CT scan revealed a thickening of the urethral wall and enlargement of the sublumbar lymph nodes (Figure 1A). At postcontrast imaging, an irregular and thickened urethral wall with a hypoattenuated center and a hyperattenuated peripheral enhancement, especially in the pelvic canal, was identified. The enlarged sublumbar lymph nodes were irregularly shaped and exhibited heterogeneous parenchyma (Figure 1B and 1C). Diagnostic cystoscopy was not available at that time.

**Figure 1.**
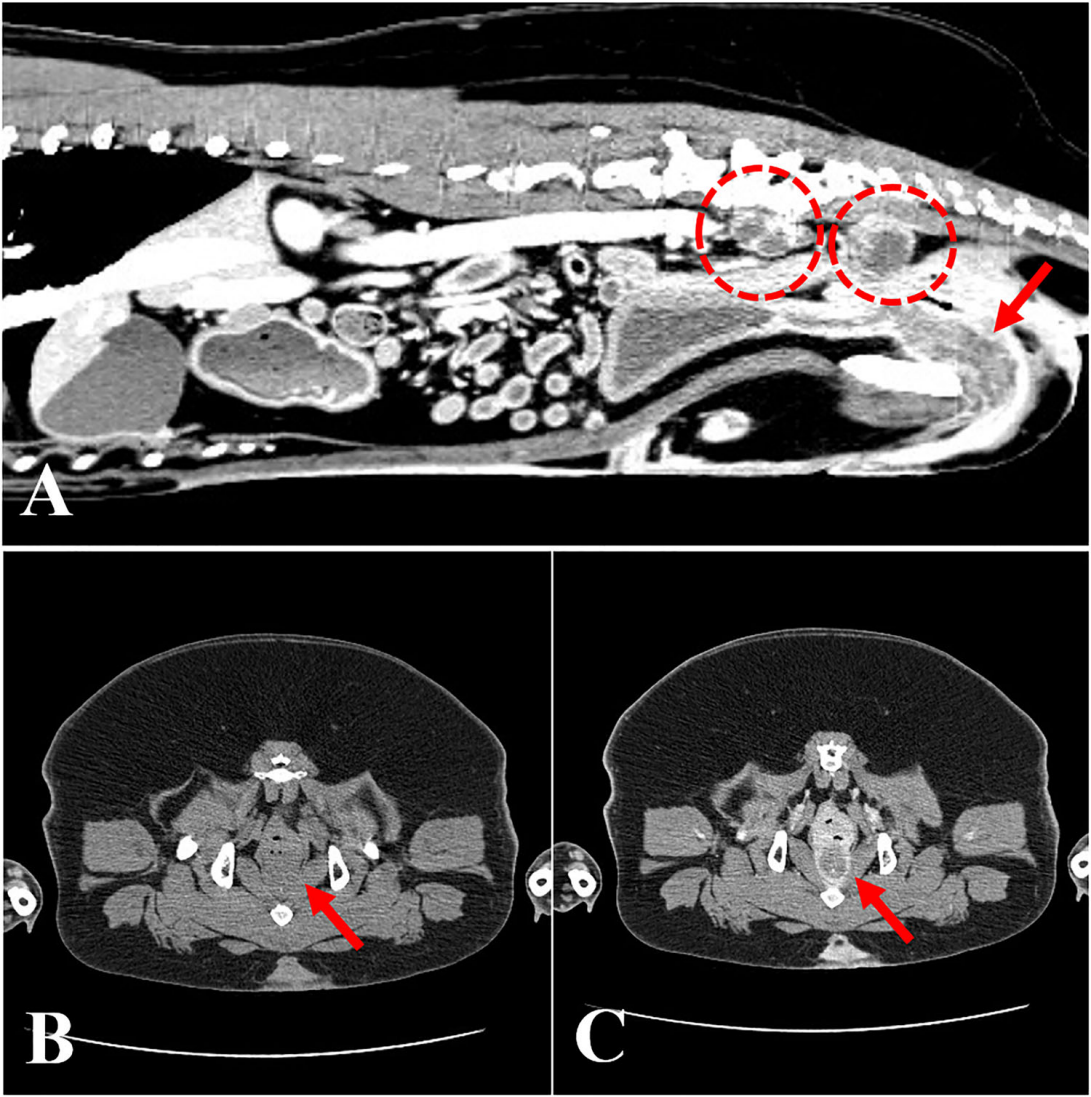
Computed tomography of a dog diagnosed with urethral transitional cell carcinoma. (A) Sagittal view revealing thickening of the urethral wall (arrow) and enlargement of the sublumbar lymph nodes (dashed circles). Note that the enlarged lymph nodes are irregularly shaped and have heterogeneous parenchyma. (B) Plain and (C) postcontrast transverse views of the pelvic region showing an irregular and thickened urethral wall (arrows) with a hypoattenuated center and a hyperattenuated peripheral enhancement.

The owner consented to the surgical excision and biopsy of the urethral tumor through cystotomy, and the dog underwent a surgical exploration of the abdominal cavity while in the dorsal recumbent position. The urinary bladder mucosal wall appeared largely normal. However, at the trigone area of the urinary bladder, a urethral mass extending to the proximal urethra was identified, and R2 resection with gross residual tumor was performed because complete surgical excision was not possible, given the location and invasiveness of the tumor (Figure 2A). The enlarged bilateral sublumbar lymph nodes were excised *in toto*. Normal and tumor urethral tissues and sublumbar lymph nodes were collected and submitted for histopathology. On serial visits, clinical and ultrasound examinations were performed, and blood (3 mL) was collected from the jugular vein in a vacutainer tube with potassium EDTA for ctDNA extraction. Plasma was immediately separated using a refrigerated centrifuge (centrifugation at 3000 g for 10 min). All samples were collected under the approval of the ethical committee of Konkuk University, Seoul (KU18141) and after the owner’s consent was obtained.

**Figure 2.**
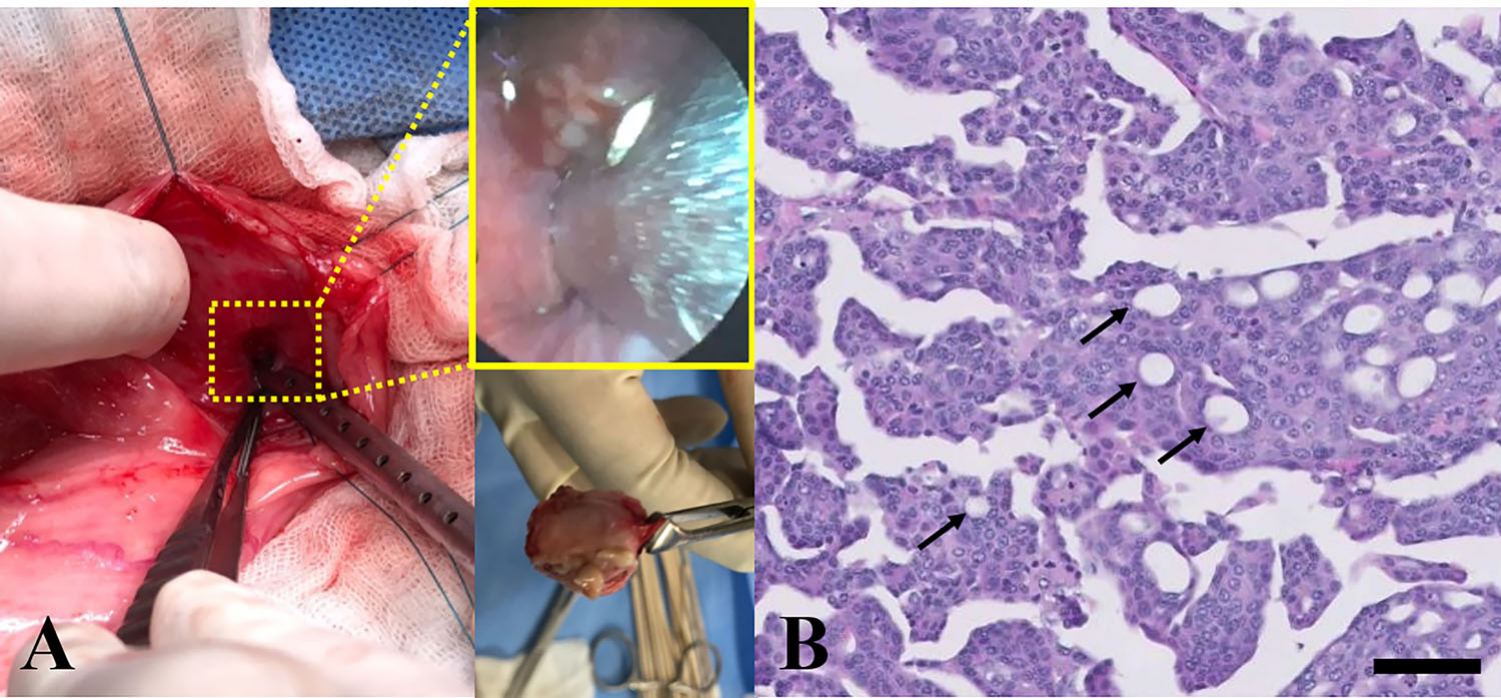
Gross morphological and histopathological features of the urinary bladder and proximal urethra in a dog with urethral transitional cell carcinoma. (A) The bladder mucosal wall was largely normal; however, a urethral multilobulated mass extending to the trigone area was identified (middle upper subset box). Bilateral enlarged sublumbar lymph nodes were excised *in toto* (middle lower picture). (B) Histopathology of the urethral mass revealed that it was composed of poorly demarcated, sessile, moderately to densely cellular, multifocally infiltrative neoplastic cells. Tumor cells were polygonal with a moderate amount of homogenous eosinophilic cytoplasm and distinct cell borders; occasionally, cells swollen due to the presence of discrete, large, clear, or eosinophilic vacuoles were observed (arrows). H&E; magnification 400×; scale bar = 50 µm. H&E, hematoxylin & eosin.

The histopathological appearance of the urethral mass sample was consistent with transitional (urothelial) cell carcinoma (TCC) (Figure 2B). Tumor cell islands, trabeculae, cords, and islands had replaced the urethral mucosa and had invaded into the submucosa and tunica muscularis; neoplastic cells are known to be associated with prominent submucosal desmoplasia. Tumor cells were polygonal, with a moderate amount of homogenous eosinophilic cytoplasm and distinct borders. Occasionally, tumor cells were swollen due to the presence of discrete, large, clear, or eosinophilic vacuoles (signet ring cells). Nuclei were round or oval and vesicular, with 1 to 3 small, but prominent, deeply eosinophilic nucleoli. Moderate anisocytosis and anisokaryosis with approximately 17 mitoses per 10 high-power fields (400×) were observed. Sublumbar lymph nodes showed normal architecture, which was nearly completely effaced by neoplastic cells that displayed features similar to those observed within the urethral mass. The bulk of the neoplasm was composed of non-papillary sessile neoplastic infiltrates that replaced the mucosa. However, an invasion of multifocal submucosal neoplastic cells associated with desmoplasia, consistent with the non-papillary/infiltrative subtype of TCC, was also detected. In the present case, neoplastic cells effaced both sublumbar lymph nodes, which was compatible with nodal metastasis. Based on these findings, the patient was diagnosed with non-papillary/infiltrative-subtype urethral TCC (clinical stage, pT2 pN1 cM0) (Mutsaers et al. 2003) with metastases to the regional lymph nodes.

Genomic DNA was extracted from surgically resected tissues using the AllPrep DNA/RNA Mini Kit (Qiagen, Valencia, CA, USA) according to the manufacturer’s instructions. Circulating ctDNA was extracted from 1 mL of plasma using the QIAamp Circulating Nucleic Acid Kit (Qiagen). Briefly, plasma samples were lysed using proteinase K and a lysis buffer and cell-free DNA fragments were bound to a silica membrane by applying vacuum pressure. ctDNA was recovered from the membrane through several washing steps.

Genotyping for the B-RAF V595E mutation was performed in tumor tissue using PCR-direct sequencing. In brief, the genomic region of canine *B-RAF* (Broad CanFam3.1, Chromosome 16:8,295,984–8,296,400) corresponding to human *B-RAF* exon 15 was amplified using specific primers (forward, 5′-ATTTCAAGCCCCCAAAATCT-3′′; reverse, 5′-GTAGCACCTCAGGGTCCAAA-3′′). The PCR products were subjected to Sanger sequencing using the BigDye Terminator v3.1 Cycle Sequencing Kit (Applied Biosystems, Foster City, CA, USA) and analyzed on an ABI 3500xl Analyzer (Applied Biosystems). All sequencing data were analyzed using the Sequencer v5.0 software.

Total RNA was extracted from surgically resected tumor and normal urethral tissues using TRIzol reagent and was reverse-transcribed into cDNA using a two-step RT-PCR kit (Invitrogen, Carlsbad, CA, USA). PCRs were run using 12-μL reaction mixtures that contained template cDNA, gene-specific oligonucleotide primers (5 pM), 100 mM of each dNTP, and 1 U of Taq DNA polymerase (Takara Bio Inc., Shiga, Japan). PCR products were electrophoresed on 2% agarose gel and visualized under a UV transilluminator. Glyceraldehyde-3-phosphate dehydrogenase (*GAPDH*) was used for the normalization of the expression levels of the target genes.

Sanger sequencing of exon 15 of the canine *B-RAF* gene revealed c.1784T > A mutation (V595E, corresponding to human B-RAF V600E) (Decker et al. 2015) in the tumor tissue and the GTG wildtype codon (no V595E mutation) in the normal tissues. The same mutation was detected in metastatic regional lymph node tissue (Figure 3). The canine B-RAF V595E mutation was monitored using circulating ctDNA from serial blood samples collected during therapy with a RAF inhibitor, sorafenib. The V595E mutation was not detected at 7 weeks after surgery, but an increase in the sequencing peak of the mutant type (V595E, c.1784T > A) was detected at weeks 11 and 12. At 16 weeks, i.e. 4 weeks after escalation to 10 mg/kg BW/day of sorafenib, the mutant sequencing peak was hardly detected and the wildtype was the dominant form (Figure 4).

**Figure 3.**
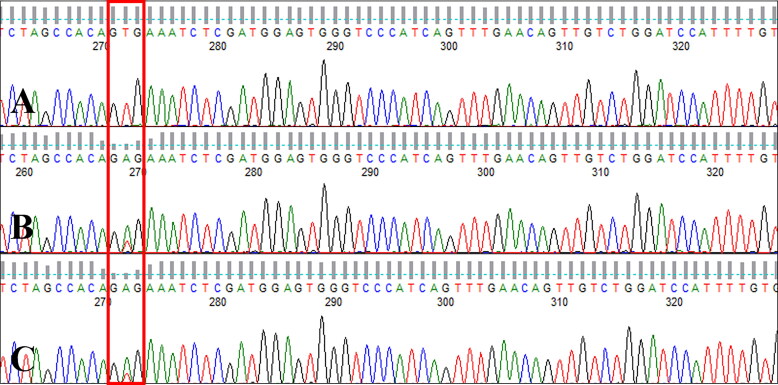
Canine B-RAF V595E mutation (human B-RAF V600E) in tumor and metastatic tissues. Electropherogram of samples from (A) normal tissue, (B) tumor tissue, and (C) metastatic regional lymph node tissue isolated from a dog with metastatic urethral transitional cell carcinoma at the first presentation day. c.1784T > A mutation (V595E) was detected in tumor and lymph node tissues (boxed), whereas the GTG wildtype sequence (no V595E mutation) was detected in normal tissue.

**Figure 4.**
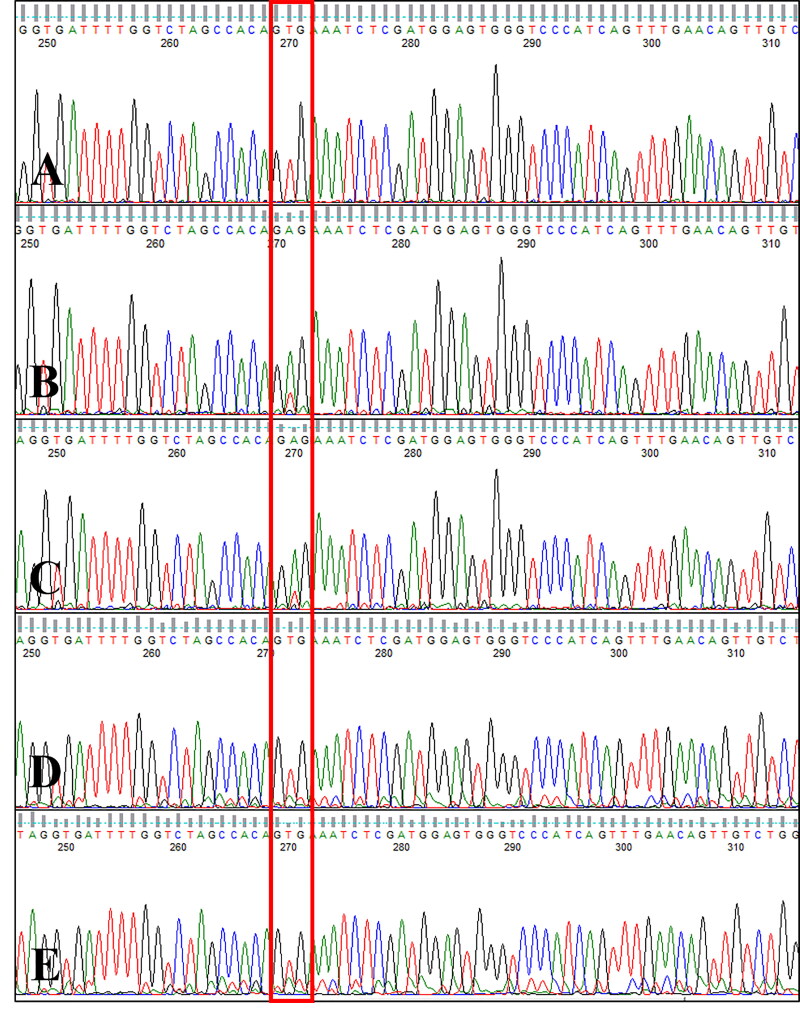
Canine B-RAF mutation status in ctDNA. Electropherogram of circulating cell-free DNA from serial blood samples collected during therapy with a RAF inhibitor in a dog with metastatic urethral transitional cell carcinoma at one week after the initiation of sorafenib therapy (A, week 7), during a gradual sorafenib dose escalation (B, week 11; C, week 12), and 4 weeks after the sorafenib dose was increased to 10 mg/kg BW/day (D, week 16; E, week 17). Note that the GTG wildtype sequence (no V595E mutation) was detected at week 7 and the mutant type (V595E c.1784T > A) was detected at weeks 11 and 12, whereas 4 weeks after the sorafenib dose was increased to 10 mg/kg BW/day (weeks 16 and 17), the GTG wildtype sequence was detected again (boxed).

The mRNA expression levels of various RTK genes, including *VEGFR-1, VEGFR-2, PDGFR-α, PDGFR-β,* anaplastic lymphoma kinase (*ALK*), epidermal growth factor receptor (*EGFR*), *ErbB2*, and *B-RAF* were compared between the tumor and adjacent normal tissues collected from the patient (Figure 5). *VEGFR-1* was overexpressed in tumor tissues, compared to that in normal tissues.

**Figure 5.**
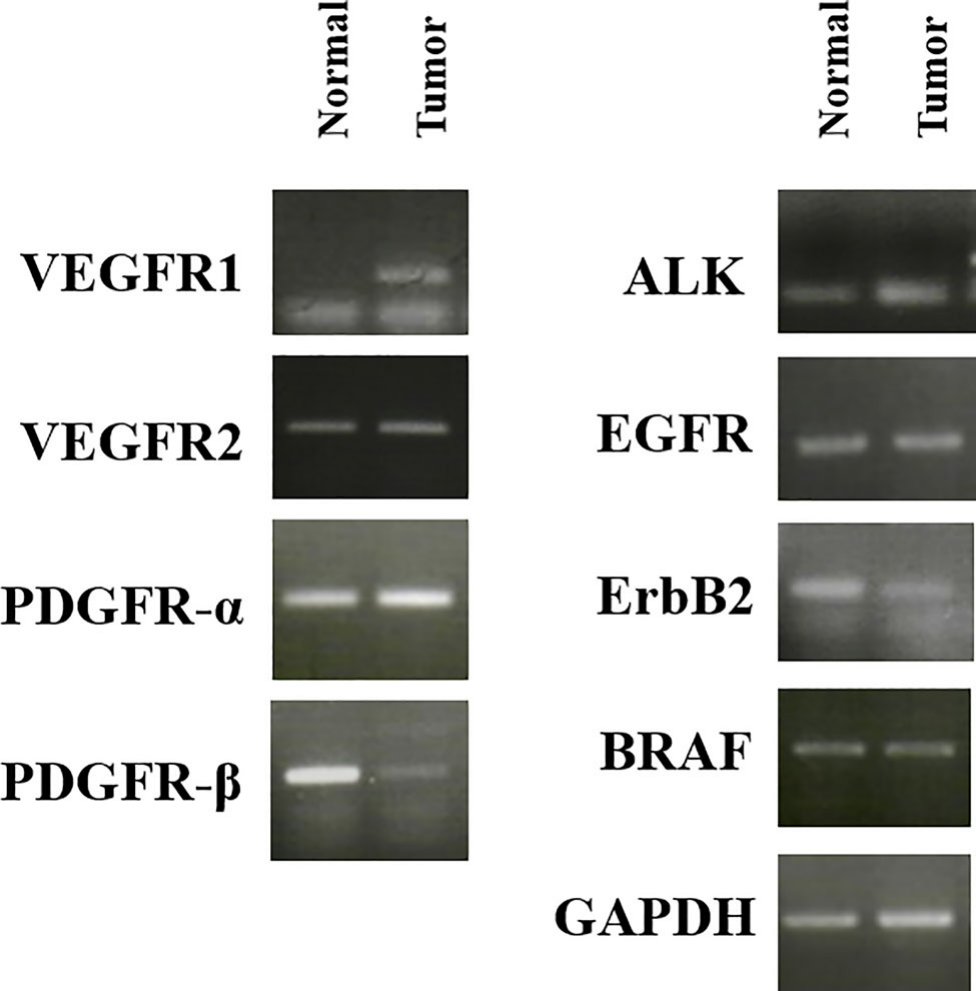
mRNA expression of various receptor tyrosine kinases in normal and tumor tissue samples from a dog with metastatic urethral transitional cell carcinoma. VEGFR was overexpressed in tumor compared to normal tissues. *GAPDH* was used for normalization. VEGFR, vascular endothelial growth factor receptor; PDGFR, platelet-derived growth factor receptors; ALK, anaplastic lymphoma kinase; EGFR, epidermal growth factor receptor.

The urethral mass of the patient was histologically confirmed as TCC of urothelial origin that was metastatic, locally advanced, and unresectable. Conventional chemotherapy (mitoxantrone, 5 mg/m^2^, IV) was initiated every 21 d for a total of 2 cycles. However, chemotherapy was stopped after the second cycle because of severe vomiting. At that time, the blood work revealed severe neutropenia (number of cells 0.432 G/L, grade IV of IV according to the VCOG-CTCAE criteria (Veterinary Cooperative Oncology Group 2004)), which was considered to be a result of severe bone marrow suppression. Although the status of the patient improved after chemotherapy cessation, the patient appeared to suffer from recurrent dysuria that was worse than before. Further chemotherapy with mitoxantrone was not provided because of critical drug intolerance.

With the consent of the owner, a combination of targeted therapy using a tyrosine kinase inhibitor and a non-steroidal anti-inflammatory drug (NSAID; piroxicam, 0.3 mg/kg BW, *per os*, q24h) was elected for third-line treatment after surgery and chemotherapy. Since tumor cells showed elevated VEGFR-1 expression as well as B-RAF mutation levels, the multikinase inhibitor sorafenib was chosen as a therapeutic agent. Starting from an oral dose of 4 mg/kg BW/day, which is one-third of the canine no-observed-adverse-effect-level(NOAEL) (Foskett et al. 2017; Wilhelm and Chien 2002), the dose was gradually escalated to up to 10 mg/kg BW/day over a period of 6 weeks. Although the B-RAF V595E mutation was not detected in ctDNA one week after sorafenib treatment initiation (week 7), the B-RAF V595E mutant ctDNA levels varied during the treatment period, as evident from longitudinal plasma samples (Figure 6). The urethral wall thickness was measured by ultrasonography (ProSound F75, Hitachi, Tokyo, Japan). To minimize measurement bias, all measurements were performed by one veterinarian and efforts were made to maintain consistency in the patient’s position and the amount of urinary bladder filling during measurements. During the gradual sorafenib dose escalation, which resulted in worsening dysuria due to increased urethral wall thickness, as indicated by ultrasonography (weeks 11, 12), the mutant sequencing peak was initially dominant; however, when the urethral wall thickness decreased and dysuria disappeared at 4 weeks after the sorafenib dose was increased to 10 mg/kg BW/day (weeks 16, 17) in combination with the NSAID, the wildtype gene became dominant again. Thus, the varying levels of B-RAF V595E mutation reflected the severity of dysuria, as ascertained based on the owner’s observations, including the observations of the frequency and duration of strained urination per day. The mutant sequencing peak tended to decrease when the clinical signs improved after the dose of sorafenib was escalated to the maximum tolerated oral dose of 10 mg/kg BW/day.

**Figure 6.**
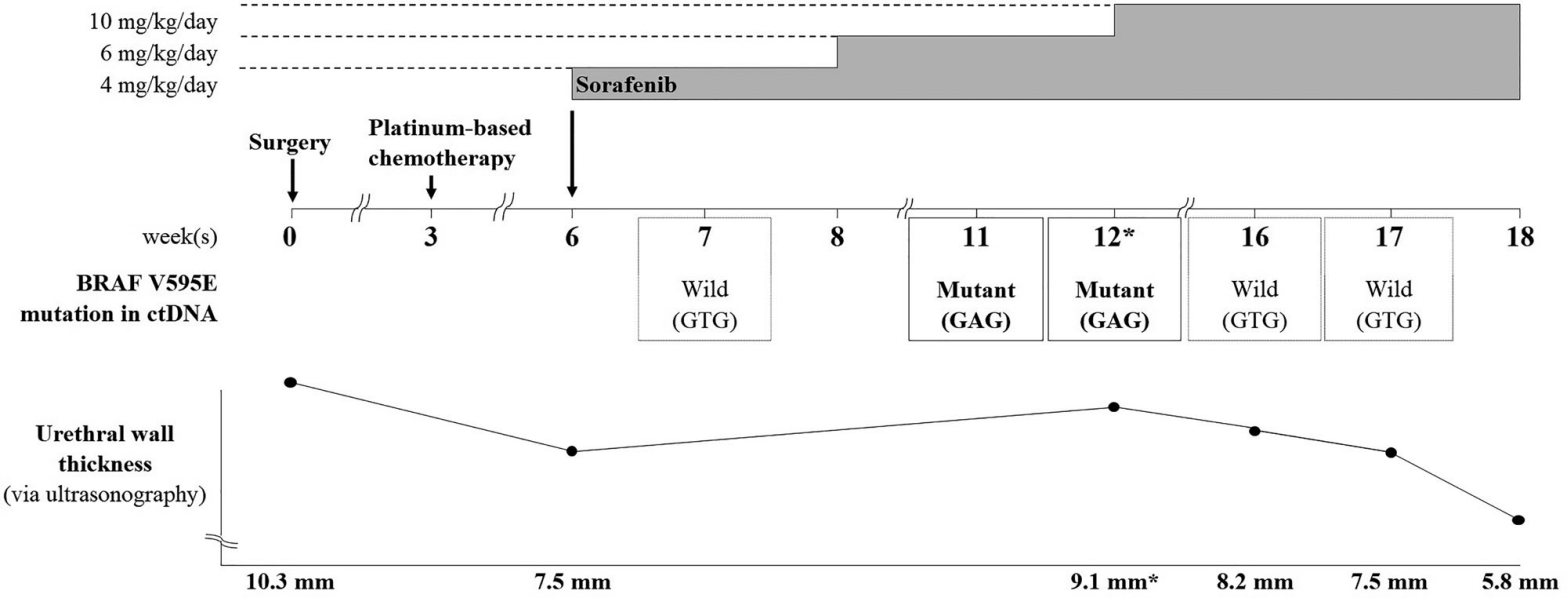
Changes in the dose of sorafenib administered and in B-RAF V595E-mutant circulating cell-free tumor DNA status according to the patient’s clinical course. Sorafenib was administered starting from a dose of 4 mg/kg BW/day, and the dose was gradually escalated over a period of 6 weeks. However, when dysuria worsened due to increased urethral wall thickness (asterisks) at 12 weeks postoperatively, sorafenib was escalated to the maximum tolerated oral dose of 10 mg/kg BW/day. Thereafter, the dysuria improved and the urethral wall thickness gradually decreased. The mutant (c.1784T > A) sequencing peak increased during weeks 11 and 12, concomitant with the worsened dysuria, although the mutation was not detected earlier (i.e. at 7 weeks after surgery). At 4 weeks after dose escalation to 10 mg/kg BW/day, the mutant sequencing peak was hardly detected and the wildtype (GTG) sequence was dominantly detected, concomitant with a significant decrease in urethral wall thickness and the resolution of dysuria.

In response to the sorafenib treatment, the dysuria was increasingly well managed for 3 months, and ultrasonography revealed a decrease in urethral wall thickness. Sorafenib was well tolerated, with a minor adverse effect (hypertension) that was controlled with ramipril at the dosage of 0.125 mg/kg BW daily, an angiotensin-converting enzyme inhibitor. The disease remained stable for more than 3 months, without any major toxic treatment effects. However, 96 days after initiation of sorafenib treatment, the dog showed a fulminant hyperexcitement reaction with excessive salivation and additional vocalization, when the owner lifted the caudal abdomen. At the time of arrival at the hospital, the patient was conscious, but the mucous membrane was pale and the patient showed panting, tachycardia, and excessive aggressiveness when manipulation was attempted. These symptoms persisted for more than 2 h, and neurologic shock caused by pain of unknown origin was suspected. Analgesics (butorphanol 2 mg/dog, 3 intramuscular [IM] injections, 15 min apart) were not effective, and anesthesia (Zoletil, 0.1 mg/kg BW, IM) was performed. Finally, the patient was euthanized. Necropsy was not performed in accordance with the owner’s request.

Transitional cell carcinoma (TCC), also referred to as urothelial carcinoma, is the most common malignancy of the urinary tract in dogs, and globally affects tens of thousands of dogs each year (Fulkerson and Knapp 2015). Due to its high malignancy, invasiveness, and metastatic potential, TCC is difficult to treat in dogs, despite the availability of intensive multimodal therapies, including surgery, chemotherapy, radiation therapy, and palliative laser ablation (Knapp et al. 2019; Weisse and Berent 2015). Particularly, in dogs with urethral TCC, complete surgical excision is unrewarding because of the location of the tumor, and thus, drugs are the main treatment modality for canine urethral TCC (Knapp et al. 2014). To our knowledge, there currently is no effective long-term single therapy for the treatment of urethral TCC in dogs; thus, the development of novel therapeutic strategies for urethral TCC, including molecular-targeted therapy, is highly anticipated. In human patients, the efficacy of molecular-targeted therapy has been shown to be limited and to depend on the presence of specific genetic alterations in cancer cells (Ishihara et al. 2019).

B-Raf (Rapidly accelerated fibrosarcoma) proto-oncogene (*B-RAF*) mutation is widely known as an important driver of the aberrant proliferation of cancer cells (Zaman et al. 2019; Sanz-Garcia et al. 2017; Ascierto et al. 2012). A B-RAF-targeting inhibitor has already been established as an important therapeutic agent in several types of human cancers (Planchard et al. 2016; Schreuer et al. 2017). Recent observations have indicated that B-RAF mutation is highly prevalent in canine TCC tumors (up to 85%) (Decker et al. 2015; Mochizuki et al. 2015). Furthermore, TCC cells overexpress vascular endothelial growth factor (VEGF); therefore, VEGF-mediated tumor neovascularization is hypothesized to also strongly contribute to the aggressive progression of TCC in humans (Al-Abbasi et al. 2009)

Microvessel density, a marker of tumor angiogenesis, has also been correlated with tumor staging, recurrence, and survival in human TCC (Dickinson et al. 1994; Jaeger et al. 1995). Elevated levels of VEGF have been correlated with TCC recurrence or progression, and increased VEGF expression in the tissues, serum, and urine of human patients with TCC has been correlated with tumor staging and prognosis (Crew et al. 1999). Inhibitors of angiogenesis have shown activity in preclinical models of TCC (Inoue et al. 2003). Sorafenib is a multikinase inhibitor with high affinity for VEGF receptors and various RAF isoforms. Accordingly, it has been demonstrated to inhibit various signal transduction pathways relevant to cancer (Wilhelm et al. 2006; Gollob et al. 2006; Liu et al. 2006). In human medicine, sorafenib has been used as a targeted therapy with clinical antitumor activity in clear-cell renal-cell carcinoma (Escudier et al. 2007), metastatic papillary thyroid cancer (Kloos et al. 2009), and melanoma (Mangana et al. 2012). Furthermore, sorafenib has been reported to be well tolerated and associated with a suggestion of clinical activity in hepatocellular carcinoma (Marconato et al. 2020) and TCC^25^ in dogs with a minimum tolerability dose of 3 mg/kg BW weekly. In the human, sorafenib dosed at 10 mg/kg BW/day (Yang and Liu 2017) noticeably targets the RAF/MEK (Mitogen activated protein/ERK kinase)/ERK (Extracellular Receptor Kinase) signaling pathway, which is considered to be important in cancer as it regulates key cellular processes relevant to the so-called cancer hallmarks, most notably, increased cell growth and survival (Chang et al. 2003). In addition to the inhibition of mitogen-activated protein kinase (MAPK), sorafenib has been shown next to target the VEGF receptor (VEGFR) also to target the platelet-derived growth factor receptor (PDGFR) (Ishihara et al. 2019; Yi et al. 2017). Further, it inhibited vasculogenic mimicry in canine mammary gland tumor cancer cells *in vitro* (Prado et al. 2019). Based on this spectrum of activities, sorafenib was selected for treatment in the present case.

B-RAF mutation analysis is a new and effective method for the confirmational diagnosis of TCC in dogs (Decker et al. 2015; Mochizuki et al. 2015). However, as tissue biopsy is an invasive process, non-invasive diagnostic methods, such as the analysis of urine containing sufficient numbers of relevant cells, as well as buffered tissue biopsy, are preferred for early analysis (Decker et al. 2015; Mochizuki et al. 2015). The use of circulating cell-free tumor DNA (ctDNA) for monitoring treatment responses has been researched in humans (Shapochka et al. 2018). So far, the presence and prevalence of B-RAF mutations have not been sufficiently confirmed in blood samples from dogs with TCC.

This study aimed to characterize receptor tyrosine kinase (RTK) expression and B-RAF mutations in a canine TCC tissue sample and to investigate the prognostic and therapeutic relevance of B-RAF mutations in ctDNA serially obtained from blood samples of a dog with metastatic urethral TCC harboring the B-RAF V595E mutation, that received third-line treatment with sorafenib. In addition, the mechanisms underlying the antitumor effects of sorafenib were investigated.

Sorafenib is a multitarget small-molecule inhibitor that impairs signal transduction by RAF-family members (Ito et al. 2017). It also inhibits the tyrosine kinase activities of VEGFR-1, VEGFR-2, VEGFR-3, PDGFR-β, Ret proto-oncogene (RET), KIT proto-oncogene (c-Kit), and Fms-like tyrosine kinase 3 (FLT3), blocking their downstream signals (Veterinary Cooperative Oncology Group 2004). The US Food and Drug Administration (FDA) has approved the use of sorafenib in human patients with liver cancer, thyroid cancer, renal carcinoma, or melanoma (Foskett et al. 2017; Mangana et al. 2012). In veterinary oncology too, a few small-molecule inhibitors have been investigated (Foskett et al. 2017). The only FDA-approved inhibitor, toceranib phosphate, has been labeled for the treatment of mast-cell tumors, although it has already been used for the treatment of other tumors (London et al. 2012), such as apocrine gland anal sac adenocarcinoma, metastatic osteosarcoma, thyroid carcinoma, head and neck carcinoma, and nasal carcinoma, owing to its multiple-target action (London et al., 2009). These targets include split-kinase family members, such as VEGFR, PDGFR, c-Kit, colony stimulating factor 1 (CSF1), and FLT3 (London et al., 2009). Unlike sorafenib, toceranib does not target the RAF/MEK/ERK pathway, making it another attractive investigational drug for veterinary patients (Foskett et al. 2017).

TCC tumors commonly harbor driver mutations in RAF/MEK/ERK pathway genes that contribute to the aggressive proliferation of cancer cells (Zheng et al. 2014). Moreover, TCC cells secrete several growth factors or cytokines, such as VEGF, to establish a suitable microenvironment for cancer progression (Al-Abbasi et al. 2009). Therefore, sorafenib might be a viable option for the treatment of TCC. Because VEGFR overexpression as well as a B-RAF mutation were observed in our canine patient with TCC, the potential antitumor effects of sorafenib were evaluated.

Recent studies have revealed that 65% to 85% of canine TCC and prostatic tumors carry B-RAF mutations (Decker et al. 2015; Mochizuki et al. 2015; Grassinger et al. 2019). Evidence for downstream pathway alterations in dog bladder cancer cells includes the dysregulation of the proximate pathway, including high levels of phosphorylated MAPK kinase (known as MAP2K/MEK) (Decker et al. 2015). Accordingly, the mutant B-RAF inhibitor vemurafenib has shown good response rates in dogs with bladder cancer (Patrawala and Puzanov 2012). The levels of the B-RAF protein and B-RAF mutations might be important predictors of the sensitivity to sorafenib in canine TCC *in vitro*. The results of this study showed that the *B-RAF* mRNA level in tumor tissues was similar to that in normal tissues. In addition, the presence of B-RAF mutation was confirmed in the tumor tissue of the patient at one point in time and in ctDNA samples obtained when the clinical signs deteriorated, whereas it could not be detected when the patient showed no clinical signs after starting therapy with the RAF inhibitor in combination with an NSAID. In contrast to previous studies, in which treatment responses were evaluated on clinical observations alone, in the present study, genetic testing of serial blood samples was helpful to monitor the therapeutic outcomes by kinase inhibitor. In this study, PCR-direct Sanger sequencing was used to detect *B-RAF* mutations. Sanger sequencing has been used as a gold standard to validate target mutations because of its high specificity (Mu et al. 2016). B-RAF V595E mutation detected in ctDNA by Sanger sequencing reflects the state of the primary tumor (Tsao et al. 2015).

VEGF secreted from cancer cells induces vigorous tumor neovascularization and thus, enables aggressive cancer growth (Onoda et al. 2014). Moreover, VEGF enhances the vascular wall permeability of the existing vessels, thereby facilitating the migration of cancer cells into the vessels (Ferrara et al. 2003), a fundamental step in metastasis. In the present study, the *VEGFR* mRNA level was increased in the tumor. The clinical resolution in our patient provides a rationale for clinical trials of sorafenib and NSAID in dogs with TCC. The correlation between VEGFR overexpression and the efficacy of sorafenib will have to be confirmed in further clinical trials.

Preliminary studies in dogs have shown that sorafenib is well tolerated (Foskett et al. 2017). In human patients, the most common adverse events associated with sorafenib treatment include cutaneous skin reactions, diarrhea, and fatigue (Cheng et al. 2009; Lee et al. 2009). In healthy female beagle dogs, mild side effects included diarrhea, reduced body weight, and emesis; however, when the doses were reduced to below 10 mg/kg BW/day, no significant side effects were observed (Wilhelm and Chien 2002; Poddubskaya et al. 2018). Foskett et al. (2017) also reported the tolerability of sorafenib in dogs with cancer, including three dogs with TCC. In their study, sorafenib administrated at 3 mg/kg BW on a once-a-week basis was well tolerated and associated with a suggestion of clinical activity, and none of the patients showed any evidence of adverse events (Foskett et al. 2017). In the present case, sorafenib was well tolerated up to the time point of euthanasia because of unexplained pain. Since the necropsy was not performed, it was not known whether the cause of the pain is due to the local tumor infiltration of lumbar spinal cord. The present findings suggest that TCC cells or tumors harboring B-RAF mutation might be a viable target for treatment with sorafenib and NSAIDs.

In conclusion, this study demonstrates the presence of a B-RAF mutation in a dog with spontaneously occurring urethral metastatic TCC with B-RAF V595E mutation. Furthermore, PCR-direct sequencing was shown to be potentially appropriate for the detection of the mutation in ctDNA, and B-RAF V595E in ctDNA may serve as a noninvasive longitudinal biomarker in canine TCC patients. Further large-scale prospective clinical research is necessary to evaluate the means by which such genetic analysis can be used for the early detection of canine TCC. The results of this study suggest that sorafenib may show therapeutic potential in dogs with B-RAF V595E-mutant TCC, particularly in VEGFR-overexpressing cases, although the survival time after the initiation of sorafenib was rather short in the current case.

## Data Availability

The data that support the findings of this study are available from the corresponding author upon reasonable request.
